# The Meanings of Smoking to Women and Their Implications for Cessation

**DOI:** 10.3390/ijerph120201449

**Published:** 2015-01-27

**Authors:** Lorraine Greaves

**Affiliations:** British Columbia Centre of Excellence for Women’s Health, E311-4500 Oak Street, Vancouver, BC V6H 3N1, Canada; E-Mail: lgreaves@cw.bc.ca; Tel.: +1-604-875-2633; Fax: +1-604-875-3716

**Keywords:** smoking cessation, women-centred approaches, gender

## Abstract

Smoking cigarettes is a gendered activity with sex- and gender-specific uptake trends and cessation patterns. While global male smoking rates have peaked, female rates are set to escalate in the 21st century, especially in low and middle income countries. Hence, smoking cessation for women will be an ongoing issue and requires refreshed attention. Public health and health promotion messages are being challenged to be increasingly tailored, taking gender into account. Women-centred approaches that include harm-reduction, motivational interviewing and trauma-informed elements are the new frontiers in interventions to encourage smoking cessation for women. Such approaches are linked to the meanings of smoking to women, the adaptive function of, and the overall role of smoking cigarettes in the context of women’s lives. These approaches respect gender and sex-related factors that affect smoking and smoking cessation and respond to these issues, not by reinforcing destructive or negative gender norms, but with insight. This article discusses a women-centred approach to smoking cessation that could underpin initiatives in clinical, community or public health settings and could inform campaigns and messaging.

## 1. Introduction

Smoking cigarettes remains one of the key global health risks for disease and premature deaths [1]. Global rates of male cigarette smoking have peaked and are declining [2], while female global rates are set to escalate dramatically in this century. Hence, responding to, preventing or treating smoking in women takes on a high priority in public health and health care. Both sex- and gender-related factors affect women’s smoking and the diseases that result [3,4,5,6,7]. Understanding the meanings and functions of women’s smoking offers insight into how best to intervene. Women-specific and women-centred approaches are gaining ground, in a bid to respond to the gendered meanings of smoking, and to tailor treatment initiatives more specifically with women in mind. These approaches include recognizing the adaptive function of smoking in women’s lives, the influence of gendered tobacco industry marketing, and specific contextual issues that contribute to women’s smoking such as trauma and violence, poverty, identity formation and social functions [8,9,10]. They also recognize some of the sex and gender specific challenges to cessation that many women face, such as biological differences in level of dependence, hormonal influences, lack of social support, high caregiving pressures, influence of partner smoking, co-occurrence of depression and mental health issues, and the experience of stigma (particularly among pregnant women and mothers) [9]. Focusing squarely on women’s lives and women’s health in these ways is a useful touch point in refreshing and improving the health care and public health response to women’s smoking. In this article a selection of qualitative research on the meanings of women’s cigarette smoking offers a basis for revisiting and potentially expanding approaches to designing treatment and offering interventions.

### When and How did Women Start to Smoke?

In most Western populations, smoking manufactured cigarettes among women emerged some years after smoking cigarettes among men had taken hold [11]. These gendered patterns of uptake were also reflected in consumption levels and prevalence rates of smoking among women with women generally smoking fewer cigarettes per day than men [12], and, with few exceptions, female prevalence rates usually peaking lower than male prevalence [11]. Some reasons for this gendered lag include social processes such as: proscriptive norms against smoking uptake by women, ascription of negative cultural stereotypes to women who smoke, and a strong socially messaged incompatibility with women’s reproductive roles. Despite such powerful social barriers, consistent and powerful gendered messaging and marketing by tobacco industries over the past century has led to the pervasive uptake of smoking among girls and women in middle and higher income countries [13,14].

The tobacco industry played a key role in generating these markets among girls and women and continues to do so in low income countries where women’s cigarette smoking rates are still low. The industry focused on creating cultural meanings attached to women’s smoking, and artfully manipulated these from decade to decade [15,16]. In so doing, the tobacco industry overrode the powerful social and cultural pressures against women’s smoking and introduced smoking and new products to girls and women [13,14]. In addition, and more pervasively, the industry succeeded in changing gender roles and norms through the use of tobacco in a range of ways. Specifically, the industry designed a series of messages over the decades of the 20th century, aimed at populations in high income countries, to shift the cultural and psychosocial meaning of women’s smoking from a negative to a positive and used these messages in their advertising and marketing, product development efforts and imagery [17]. This gendered approach to smoking behaviour and uptake was very successful in these contexts, changing the cultural meaning of smoking from negative associations such as prostitution, manliness and rebellion, to positive images of independence, success, health and self-care [13,15] over the course of several decades. These cultural meanings of smoking to women, while generated externally, eventually affect the psychosocial processes of creating meaning by women who smoke, and become internalized [18].

Ultimately these practices, themes and images associated with women’s smoking were to be challenged in high income countries, but not until long after significant damage was done to women’s health [4,13,19,20,21]. However, in this century, similar targeted techniques are being used to involve women in low income countries in smoking cigarettes, with ethnoculturally-specific marketing and advertising to women (*i.e.*, the Virginia Slims “Find Your Voice” global campaign: [13]). In addition, tobacco growing and cigarette production has relocated, or is relocating to low income countries, and many women and children are laborers in tobacco growing and production activities [22]. These economic activities engage and change the local economies in ways that render smoking and tobacco growing and production increasingly important, and more difficult to resist. These activities directly contribute to the engagement of new and large populations of women in countries such as India and China, and regions such as Africa, where women’s use of cigarettes remains at low levels [23]. Significant growth and profits for the industry exist in these locations where comparatively under-resourced responses from societies and governments remain the norm.

Ironically, and sadly, health system and health promotion efforts addressing cigarette use in the past 50 years in western countries did not adopt a similarly marked gender analysis and did not investigate women-specific and context specific approaches or messages, despite the stark example of success of this approach by the tobacco industry [15,20]. In fact, the early responses to women’s smoking in high income countries were focused solely on the effects of smoking on women’s reproductive abilities, and usually conflated “women’s smoking” with “pregnancy and smoking” [15,20,21,24]. This conflation persisted for some time and meant that attention to *women’s health* was overlooked or missing, women were viewed only as vessels affecting fetal health, and as a result, any cessation that occurred during pregnancy was generally short lived and led to relapse [8]. Other than this extraordinary attention to pregnancy and smoking, little gendered or sex-specific research or programming emerged until well into the 1990s [19,20,25]. This was a lost opportunity and delayed any thoughtful gendered responses to smoking among women that ought not to be prolonged in this century in high income countries or, mistakenly repeated in low income countries in the 21st Century.

At the same time, the patterns of smoking among women in high income countries, while generally declining, are doing so in inequitable ways. Social and economically marginalized women and girls, Indigenous women and girls, those with histories of violence, mental health problems and other addictions, and those living in poverty with burdens of care giving and stress are most likely to be new and persistent smokers. These vulnerabilities to taking up or continuing the use of cigarettes are increasingly well-documented [26,27,28,29,30,31,32,33,34,35,36,37,38,39,40]. It is these vulnerabilities and population-specific uses of cigarettes that concern us now, along with rapidly growing rates in new regions of the world. Within these population-specific rates are embedded numerous lessons, some of which may be useful in addressing or short-circuiting the emerging and rising rates of women’s smoking in low income countries.

These vulnerable groups of women who still begin or continue to smoke in high income countries after general population-level declines have taken place, may offer us valuable insights into key issues in assisting women with cessation, or generating social and cultural responses to women’s smoking that will help women either resist uptake or manage cessation. These groups in high income countries that are vulnerable to smoking are often marginalized, of low socioeconomic status, are indigenous, have experienced trauma, violence or mental health issues, other substance use issues [26,41]. One avenue to understanding the persistence of smoking in these settings is through analyses of the meanings of smoking to women.

## 2. Meanings and Functions of Women’s Smoking

Qualitative investigations into smoking among women, while relatively few in number, have been carried out in recent decades, drawing attention to identifying the meanings and functions of women’s smoking and to assess how smoking fits into, or reflects women’s lives. These research approaches have led to deeper understandings of women’s smoking, and to adjustments or new responses to prevention and cessation. This section addresses some key themes in those investigations with a range of groups of women, including post partum, low income, adolescent, abused women, feminists, and women with chronic obstructive pulmonary disease (COPD).

Some research on meanings include my previous work, published in 1996, where I identified five major themes for women in Canada and Australia who smoke by conducting interviews with women who had experienced abuse, indigenous women and women who self-described as feminists, to generate a range of perspectives on the meaning of smoking in their lives [15]. For all of these women, there were five main themes: organizing social relationships, creating an image, controlling emotions, exercising dependency and creating identity [15].

All of these meanings have various manifestations. For example, using smoking to bond or repel others in social interactions can be either a lubricant or an equalizer in social settings or, in some cases, a barrier or “distance” in relating to certain people or remaining in threatening, risky or boring situations. Two women illustrate this:

*“I’ve felt very much at war with that role (mothering) since day one. (I was) using cigarettes to create a sense of space around myself, to mark time for myself, to mark time out for myself, to mark a sign to be isolated.”*.

(Alexia, quoted in [15] p. 47)

*“I could hide, by looking down, or behind the smoke…I could hide my fear of him.”*.

(Victoria, quoted in [15] p. 48)

The issues surrounding image creation are perceived to be directly attributable to the efforts of the tobacco industry, and indeed, women report elements of risk-taking, rebellion, differentiation from others or independence in their self-images, an echo of many of the advertising themes directed at women in the 20th century. For example:

*“I just wanted to be cool, being cool was always important to me in the rebel part of me. It was also wrong, so I did it*.*”*.

(Alberta, quoted in [15] p. 50)

Controlling emotions via smoking cigarettes is an interesting element as it offers insight into women’s roles and experiences, often manifesting in using cigarettes to “suck back anger” or suppress boredom, tension, stress or irritation.

*“I don’t want to be miserable, I don’t want to bark at people. Because I like to be nice.”*.

(Vera, quoted in [15] p. 57)

*“When we (my husband and I) were happy I didn’t smoke much but when things went bad I smoked twice as much.”*.

(Jessie, quoted in [15] p. 60)

Exercising dependencies through smoking include reflections on cigarettes being “best friends”, sources of dependable comfort or support that offer anchorage and a source of control for women.

*“My cigarettes have been more consistent than any people in my life.”*.

(Carla, quoted in [15] p. 63)

*“They’re (cigarettes) like a partner. They’re the most dependable partner I’ve had. Cigarettes are my best friend…they’re the most dependable thing, its frightening to think of it.”*.

(Alberta, quoted in [15] p. 65)

All of these meanings suggest that smoking cigarettes functions in some very practical ways for women, particularly for women in difficult life circumstances. Cigarettes assume the role of friend and offer solace and comfort. But the identity issues surrounding women’s smoking are perhaps the most intricate and deep, and offer insights into how complex and nuanced smoking’s meanings can be. The essence of smoking for women is that smoking offers a route to controlling various emotions, images or circumstances, but, as its addictive qualities increase, smoking itself controls and is controlling of women.

*“Cigarettes do control me, I guess, but I control them*.”.

(Barb, quoted in [15] p. 72)

This contradiction is an essential aspect of understanding women’s smoking. As much as the introduction to and early stages of smoking offer a welcome option for women in reducing or recalibrating their emotions and repairing or refreshing their lives in some way, by the time smoking is a dependency or an addiction, it controls women’s options for change, health and identity, and often prosperity.

Hilary Graham’s research on smoking with disadvantaged women in the United Kingdom offers some insights into the practicalities of smoking’s function for women who experience a series of stressful and negative events and experiences in life [25]. In the early 1990s, Graham conducted interviews with 905 women with children living in manual occupation households. She reports that women entrenched in disadvantaged circumstances use smoking to cope with their lives in a range of ways such as providing a break from the pressures of care-giving or other forms of work, relieving stress, or instituting distance between them and their children’s demands [25]. One example Graham reports suggests that smoking prevented lashing out at a child:

*“Sometimes I put him outside the room, shut the door, and put the radio on full blast and I’ve sat and had a cigarette, calmed down and fetched him in again.”*.

(Mother with pre-school child quoted in [25] p. 93)

Similarly, another UK report on low income women and smoking [42] illustrates the small territory that some women have in which to carve out “space”:

(Smoking is) *“the only thing I do for myself, isn’t it? I have to do things for the baby and my husband, but smoking is about the only thing I can do for myself.”*.

(Young mother, quoted in [43] p. 2)

Bobbie Jacobson’s 1986 book *Beating the Ladykillers: Women and Smoking* [21], was instrumental in bringing women’s smoking to world attention and beginning to frame women’s smoking as a feminist issue, deserving of a different kind of attention than that normally given. She conducted interviews with women in the UK and offers some telling quotes about how the women she interviewed interpreted their smoking. Some of these reflections were extremely practical:

*“I can remember when the children were very young, I learned that if ever I sat down, they immediately came and crawled all over me. So to try and have some peace, I used to prop my library book on the top of the black fireplace, and have my cup of tea and cigarette standing up. That was the only time if felt I’d got some space on my own….that was the real highlight of the day.”*.

(Viv, quoted in [21] p. 93)

Other researchers have also investigated meanings, such as those associated with adolescent smoking [44,45], suggesting that these contradictions develop fairly early and quickly in smokers. Tilleczek and Hine [46] reported findings from their interviews held with a sample of 20 younger (12 year old) and older (16 year old) adolescents who smoke in northern Canada and noted the following among the females: *“For the youngest women, the positive valence was related to the socially adaptive properties such as gaining acceptance with the older crowd, attracting the opposite sex, fitting in, rebelling, and having fun (playing)”* ([46] p. 280). The older female adolescents were more reflective about addiction, and identified control as a meaning of smoking [46]. All of the females were *“frequently articulate about the place of smoking in meeting a need to fit in with and/or rebel against their social contexts”* ([46] p. 283]), reflecting the contradiction that smoking presents. Many of the adolescents reflected on the stigma that they already experienced as smokers and how much they notice it. These insights with adolescents suggest that reflections and insights into the meaning of smoking begin early, and could be elicited and capitalized on by practitioners as openings for conversations about smoking.

Sometimes these interpretations of smoking remain strong, even in the face of acquiring a serious smoking related illness such as COPD. Jonsdottir and Jonsdottir [47] report findings from interviews held with a small group of seven Icelandic women smokers between the ages of 47 and 65 years who had been hospitalized for COPD, many of whom had quit and relapsed. They report smoking for these women as like a “spider’s web” that controlled them even though they felt intense shame and guilt about continuing to smoke in the face of a serious smoking-related lung disease. The women often felt that the utility of smoking in helping them deal with their life problems and issues, particularly stress, mixed with the addiction to nicotine, prevented them from quitting and staying quit. These sentiments speak to the tenacity of the meanings and functions of smoking for women, persisting for some even in the face of serious, chronic, disabling disease.

These selected examples from the literature on meanings of smoking for women allows us to gain insight into the meanings of smoking for many women. The details they offer on how smoking functions in daily life for many women points to some of the issues and approaches required from smoking cessation interventions. These functions appear to be largely adaptive: offering solace in face of difficulty, dangling a symbol of better things, or serving as a solution to everyday circumstances that may be difficult or may need enhancement. Understanding these aspects of cigarette smoking is essential for acknowledging these elements, and developing appropriate responses in cessation interventions or health promotion campaigns. Certainly, the tobacco industry has understood these women-specific concerns and desires for some time, and has harvested considerable profit from this understanding. It is important to consider how a health-oriented response might look.

## 3. What Approaches Might Work?

Typical mainstream cessation advice often focuses on brief interventions or standard tips for quitting [48,49]. One common, tested approach is the 5As, which underpins many cessation programs or is used in the context of other health promotion or health care programming, such as prenatal care [50]. Nicotine replacement therapy (NRT) is also a low cost and useful cessation aid, in some jurisdictions supplied to low income smokers at reduced or no cost [51,52]. Cessation advice and assistance are part of comprehensive tobacco control, which also includes surveillance, policy, taxation and pricing initiatives, advertising and promotion bans, prevention and reduction of exposure to secondhand smoke [53]. Comprehensive tobacco control has been largely successful in high income countries in addressing and reducing prevalence of smoking across populations. But these declines have often masked high rates of smoking in some specific vulnerable groups of women (and men). In response, tobacco policies and programs have been challenged to respond in more tailored ways to certain vulnerable sub populations in recent years [26,28,29,39,54,55].

There have also been increased efforts to address the social context of women’s smoking, such as the impact of partner smoking during pregnancy [56], the impact of household members’ smoking in the context of reducing exposure to smoke in the home [57,58,59], the links between smoking and experience of intimate partner violence [60] and mental health issues [61], other substance use [62,63] and the links between employment roles and smoking [35,64,65]. Similarly, the understanding of vulnerability and social context to better understand men’s smoking is growing [66,67].

In addition, and useful to all genders, increased demands in broader health research to take sex and gender into account has assisted in drawing attention to the impact of both sex and gender on health [68] and also drawn more attention to gender, tobacco and health [4] including the effects of gender on women’s (and men’s) smoking [67,69].

This growing attention to sex, gender and social context is welcome, and offers an opportunity to more closely link meanings and functions of women’s smoking to potential responses. Various literatures that address women’s health, recognize the impact of social context, gendered trauma and violence, higher rates of poverty, and high care-giving pressures and offer some ideas about what these issues might mean for cessation responses and messages. Linking the meanings of smoking to women to these issues and contexts in a direct manner may help in addressing smoking cessation for women in more realistic ways. Indeed, more qualitative research, engagement with cigarette smokers, their families, households and communities and co creating cessation initiatives are all measures that could be useful going forward.

Women-centred care is a concept that addresses women’s health in the context of their lives [70]. This means that women’s experiences are taken into account and factored into understanding their health behaviours. For example, women who experience intimate partner violence (IPV) are more likely to smoke [60] and women who have a range of mental health concerns are more likely to smoke [71]. These overlaps not only indicate how smoking functions, but also indicates how health care providers need to have a more comprehensive understanding of women’s circumstances and experiences as they respond to women’s smoking.

This recognition of social context also demands that we engage with approaches such as harm reduction and trauma-informed initiatives. In the context of tobacco cessation, harm reduction might mean that we may discuss gradual reduction, reduction of exposures to others’ smoking, improving other aspects of health, such as nutrition and physical activity, or even recognizing that a woman is not ready to quit at this moment. To be trauma-informed means that we implicitly recognize the impact of trauma and pervasive violence in many women’s (and some men’s) lives [72] and explicitly avoid judgmental, punitive, or shaming approaches to addressing women’s smoking that may be re-traumatizing, and close the door for reflection and development of insight. Being trauma-informed is a stance that providers and systems can take, that is not dependent on disclosure of trauma by individuals [73].

Research has identified four main principles in women-centred care for tobacco use [9]. First, women-centre care is tailored specifically for women, including taking into account biological and psychosocial factors affecting care and intervention. Second, women-centred care builds confidence and increases motivation, through identifying barriers to change, as well as opportunities and enacting stigma reduction and social support. Third, women-centred care incorporates social justice issues, such as recognizing that other socioeconomic concerns such as violence and poverty also factor into decision making about tobacco use and health. And finally, women-centred care is comprehensive and holistic, focusing on the entire context of a woman’s life and drawing together treatment for other issues such as alcohol use, mental health issues, or trauma.

Some innovative interventions with low income single mothers directly address the impact and pressures of care-giving and offers harm reduction tips such as considering how to choose a place to smoke to reduce smoking and reduce children’s exposure to smoking [74]. These approaches recognize the realities of being lone mothers who smoke, address immediate child health concerns, while offering support, space for change, and an opportunity to build on strengths to develop insight among single mothers who smoke. Interestingly, although not a cessation program, the majority of the women who participate in a program such as STARSS end up trying cessation [74].

Similarly women-only group interventions aimed at smoking reduction and/or cessation may discuss a range of other components such as self-care, violence in relationships or nutrition and offer supports such as transportation and child care assistance [75,76]. Groups such as these can create the support for gradual changes, and offer social networks to assist [76,77]. A group program developed in Northern Ireland aimed at disadvantaged women called *Stopping for Me*, divided its program into a three week course that created space for women to discuss their smoking freely, in a supportive atmosphere. This section included reflections on smoking, their relationship to the health system, impact of advertising and family histories. This space generated initial reflections on smoking as one of many women’s health concerns, but also recognized the effects of structural factors such as health care and tobacco industry advertising. The facilitators reported that this phase could often give rise to anger at these external factors, which was important in diverting the blame from women to larger pressures [78]. The second phase, not necessarily taken up by all the women in phase one, was a 12 week cessation oriented program where the women were in control of the agenda that included sessions on conflict resolution, stress, and coping. There was no pressure to quit, however, and the women reported that this was their first positive experience with cessation [78].

A similar approach was integrated into a residential women’s addiction treatment centre in Canada [79]. In this setting women were offered the opportunity to name their most problematic drugs, and often named nicotine as one of their top three, alongside cocaine and alcohol. They were then given opportunities to address their smoking in an integrated manner, and asked to reflect on three themes: the health effects of smoking on women; the links between smoking and victimization, trauma and coping, and the effects of tobacco advertising aimed at women, [79]. These collaborative, non-threatening approaches created a non judgmental atmosphere, and integrated a discussion and response to smoking into a treatment environment that had previously ignored tobacco use among its clientele.

For mainstream health care practitioners there are many opportunities to increase their effectiveness in dealing with women’s smoking. But shifting from “telling” to “asking” is an important aspect of this. There are models for intervention for health care providers to invite openness to counseling and to address difficult substance use issues with women [80]. For example, the typical 5As approach [49] can be augmented with a trauma-informed, motivational interviewing approach [81]. Motivational interviewing encourages a conversation about smoking, keeps it aflame at each encounter and moves it toward points of insight and change that women, not practitioners, can claim and control [82]. A Cochrane systematic review of motivational interviewing (MI) for smoking cessation revealed modest but significantly greater rates of smoking cessation among participants receiving MI compared to brief advice or usual care [83]. Practitioners using these approaches act as partners in change, assisting with the provision of information and recognizing that building toward change is a matter of building confidence and working through ambivalence. These interactions need to be trauma-informed, recognizing that may women experience trauma, whether revealed or not, and benefit from safe, trusting, non-punitive and non-authoritarian approaches. Again, these approaches are more about “what happened to this woman?” as opposed to “what is wrong with this woman?”. Detailed approaches to a brief intervention using these approaches, with explicit advice for practitioners on how to phrase their questions are available [9]. Trauma informed practice for women with co-occurring substance use and mental health disorders has been found to decrease substance use, depression and trauma symptoms [84,85].

All of these innovative approaches link directly to the meanings and functions of smoking that women report and respect and build on them to obviate the “benefits” of smoking for women as adaptations to circumstances and balance them against the “costs” [15]. Indeed, decision-making tools encourage women to assess their smoking against these two elements, in order to clarify and identify points for change [9].

Social context and women-specific and women-positive understandings of smoking are also important in health promotion. In mainstream health promotion activities, gender has long been absent [86] and attempts to integrate gender have often fallen short by generating messages or programs that play on destructive gender stereotypes or norms and focus on individual behaviour change as opposed to recognizing the effects of social context [87]. In smoking cessation interventions, there are past and current examples of tobacco campaigns or messages that do this.

For example, a UK poster decrying smoking during pregnancy in 1973 generated outright shame and guilt by featuring a naked pregnant woman with the caption: *“Is it fair to force your baby to smoke cigarettes?”* [88]. An American poster decrying the negative effects of smoking focused on the effects on women’s beauty by picturing an elderly woman with extreme wrinkles and an unkempt appearance [89] An image in a poster in India from 2008 bizarrely features a burning cigarette as a replacement for a woman’s nipple on a naked breast [90] to imply that women who smoke and breastfeed are forcing their infants to smoke.

These messages all rely on and reinforce negative gender stereotypes about women’s roles and value (such as physical appearance or sexual attractiveness or ability to reproduce) to elicit support for non-smoking, as opposed to positive reasons such as increased control, better health, ability to be active* etc.* Even tobacco control experts who recognize these subtleties argue that using gendered stereotypes in prevention and cessation is justifiable if they resonate with the target audience [91]. But certainly the bar on elevating and interpreting gender can be raised, and tobacco control can lead, not follow. Improvements in health promotion would not only integrate women and gender, but do so in a transformative and empowering manner. Some options for tobacco control initiatives that do this are naming women’s health and self-respect as the prime motivator for cessation, as opposed to reinforcing gendered norms of sexual attractiveness or femininity, and focusing on a full range of women’s roles in designing tobacco cessation other than reproduction and pregnancy [92,93]. The field is ripe for the development of such approaches.

## 4. Conclusions: Meanings for Intervention

It is still unclear if the four stages of the tobacco epidemic that Lopez and colleagues described in 1994 will roll out in the same ways in the low income countries in the 21st century [94,95]. Many factors have emerged or changed since the model was developed, such as increased globalization, urbanization and migration, as well as rapidly changing global communications and shifting gender norms. How these pressures will affect gender, equity and tobacco use remains to be seen [96]. Nevertheless, the prediction that worldwide cigarette smoking among women will have doubled between 2005 to 2025 [53] impels us to try to learn from past mistakes and oversights in the high income countries. Certainly, the sub populations of women who continue to smoke in high income countries, as well as the women who are taking up smoking cigarettes in low income countries would all benefit from a clearer focus on equity, social context and more nuanced responses to women’s smoking than those currently proffered in typical mainstream health care and health promotion. Whether the misplaced and misguided attempts at intervening in women’s cigarette use rampant in the high income countries become a lesson for change, or a template for mimicry in low income countries depends on our collective ability to learn and make effective and culturally sensitive knowledge translation.

A shift in emphasis in tobacco cessation approaches is required to meet the patterns of women’s smoking in high income countries and in emerging groups of women who smoke in low income countries. This shift needs to recognize the meanings of smoking to women, as well as the social and cultural contexts in which women’s cigarette smoking occurs and the functions that it performs. Historical experiences with cessation, as well as experiences in related women’s health fields such as alcohol use suggest that women-centred, trauma-informed and harm reduction oriented approaches could be useful in refreshing or augmenting the approach to women’s smoking, in any of these circumstances [73,97]. These approaches rely on suspending judgment and blame, taking the emphasis off individual behavioural change and placing the challenges of smoking cessation in the context of the social and economic pressures that women experience. It also means opening the door to conversations about smoking that do not imply a commitment to cessation, and that engage women and health care providers in a dialogue about the realities of smoking. This will require a reorientation among health professionals in patient interactions, as well as among health promotion practitioners designing messaging, campaigns or health promotion initiatives. These shifts in practice require a broader lens, tuned in to gender, gender equity and the use and encouragement of transformative and positive gender roles, as opposed to negative, punitive or limiting gender stereotypes [98].

The investigations of meanings of smoking are critically important in this shift, as they illustrate the lived experiences and interpretations of women who smoke. These experiences give rise to understanding how to intervene, and what properties of cigarette smoking may have to be replaced in a smoke free life. In my previous research, the final question I asked women who smoke was always: *“what would your life have to be like in order for you to not smoke?”* [15]. This question always elicited thoughtful, and sometimes profound sentiments, but gave clear examples of how much change is required (both internal and external) in order to reclaim a smoke free life. Tod [99] asked pregnant women about their smoking experiences and quotes one of her respondents as saying: *“To be able to stop, you’ve got to have a…a bloody good life”* [99].

Such methods might also serve as interventions. In their investigation of adolescent smokers, Tilleczek and Hine [46] suggested that research was also useful for more than gathering data: *“A further practical implication of the study is that the methodology could itself be useful in prevention efforts”* [46] (p. 285). Many women-centred interventions make such collaborative consciousness raising central to their approach. For example, Barr indicated the impact of placing women’s smoking in the context of social and economic factors in her preliminary sessions with women smokers [78]: *“Whilst political contextualization was a conscious approach taken by the project as a means of empowering women, it did not fully value the impact of women’s anger as a trigger for positive action. This proved to be a powerful way of moving on discussion from the model of woman as a victim of poor health and poor health care to one that was empowering, and that could lead to positive change”* [46] (p. 25)..

Motivational interviewing, which is the basis of the method in *Liberation!* *Helping Women Quit Smoking: A Brief Tobacco Intervention Guide* [9] also takes this approach: giving space and time for women who smoke to have their experiences and opinions heard and considered and having value attributed to those thoughts. Often, women given this opportunity to talk about meanings, either in research or in interventions settings reveal that this is the first time they have had an opportunity to consider smoking in a meaningful way and in a context with respect and no judgment. This lesson is integral for health care providers doing brief interventions, however brief. Practitioners could easily broaden their stance to offer such openings that reflect elements of women centredness, motivational interviewing and trauma informed practice, at little or no cost. Researchers investigating new models or approaches to women’s smoking cessation could prioritize community and subject engagement in designing and testing interventions. Likewise, policy and program developers, along with public health campaigners could promote transformative messages and approaches in generating initiatives with women. Giving women space to be heard about smoking may open up a process and conversation that ultimately leads to better health.

## References

[1] (2009). Global Health Risks: Mortality and Burden of Disease Attributable to Selected Major Risks.

[2] Mackay J., Amos A. (2003). Women and tobacco. Respirology.

[3] Torchalla I., Okoli C.T., Hemsing N., Greaves L. (2011). Gender differences in smoking cessation. J. Consult. Clin. Psychol..

[4] Amos A., Greaves L., Nichter M., Bloch M. (2011). Women and tobacco: A call for including gender in tobacco control research, policy and practice. Tob. Control.

[5] Hitchman S.C., Fong G.T. (2011). Gender empowerment and female-to-male smoking prevalence ratios. Bull. WHO.

[6] Sørheim I.-C., Johannessen A., Gulsvik A., Bakke P.S., Silverman E.K., de Meo D.L. (2010). Gender differences in copd: Are women more susceptible to smoking effects than men?. Thorax.

[7] Huxley R.R., Woodward M. (2011). Cigarette smoking as a risk factor for coronary heart disease in women compared with men: A systematic review and meta-analysis of prospective cohort studies. Lancet.

[8] Greaves L., Poole N., Okoli C.T.C., Hemsing N., Qu A., Bialystock L., O’Leary R. (2011). Expecting to Quit: A Best-Practices Review of Smoking Cessation Interventions for Pregnant and Post-Partum Women.

[9] Urquhart C., Jasiura F., Poole N., Nathoo T., Greaves L. (2012). Liberation! Helping Women Quit Smoking: A Brief Tobacco Intervention Guide.

[10] Bottorff J.L., Haines-Saah R., Oliffe J.L., Sarbit G. (2012). Gender influences in tobacco use and cessation interventions. Nurs. Clin. N. Amer..

[11] Lopez A.D., Collishaw N.E., Piha T. (1994). A descriptive model of the cigarette epidemic in developed countries. Tob. Control.

[12] Perkins K.A., Donny E., Caggiula A.R. (1999). Sex differences in nicotine effects and self-administration: Review of human and animal evidence. Nicotine Tob. Res..

[13] Amos A., Haglund M. (2000). From social taboo to “torch of freedom”: The marketing of cigarettes to women. Tob. Control.

[14] Toll B.A., Ling P.M. (2005). The virginia slims identity crisis: An inside look at tobacco industry marketing to women. Tob. Control.

[15] Greaves L. (1996). Smoke Screen: Women’s Smoking and Social Control.

[16] Tinkler P. (2001). “Red tips for hot lips”: Advertising cigarettes for young women in britain, 1920–1970. Womens Hist. Rev..

[17] Brandt A.M. (1996). Recruiting women smokers: The engineering of consent. J. Am. Med. Assn..

[18] Rozin P., Singh L. (1999). The moralization of cigarette smoking in the United States. J. Consum. Psychol..

[19] Greaves L. (1990). Background Paper on Women and Tobacco.

[20] Jacobson B. (1981). The Ladykillers: Why Smoking Is a Feminist Issue.

[21] Jacobson B. (1986). Beating the Ladykillers: Women and Smoking.

[22] Greaves L., Jategaonkar N., Sanchez S. (2006). Turning a New Leaf: Women, Tobacco, and the Future.

[23] Palipudi K.M., Gupta P.C., Sinha D.N., Andes L.J., Asma S. (2012). Social determinants of health and tobacco use in thirteen low and middle income countries: Evidence from global adult tobacco survey. PLoS One.

[24] Oaks L. (2000). Smoke-filled wombs and fragile fetuses: The social politics of fetal representation. Signs.

[25] Graham H. (1993). When Life’s a Drag: Women, Smoking and Disadvantage.

[26] Greaves L., Hemsing N. (2009). Women and tobacco control policies: Social-structural and psychosocial contributions to vulnerability to tobacco use and exposure. Drug Alcohol Dependence.

[27] Hemsing N., Greaves L., Poole N., Bottorff J. (2012). Reshuffling and relocating: The gendered and income-related differential effects of restricting smoking locations. J. Environ. Public Health.

[28] Greaves L., Jategaonkar N. (2006). Tobacco policies and vulnerable girls and women: Toward a framework for gender sensitive policy development. J. Epidemiol. Community Health.

[29] Graham H., Inskip H.M., Francis B., Harman J. (2006). Pathways of disadvantage and smoking careers: Evidence and policy implications. J. Epidemiol. Community Health.

[30] Graham H. (2009). Why social disparities matter for tobacco-control policy. Amer. J. Prev. Med..

[31] De Finney S., Greaves L., Janyst P., Hemsing N., Jategaonkar N., Browne A., Devries K., Johnson J., Poole N. (2013). “I had to grow up pretty quickly”: Cultural and gender contexts of aboriginal girls’ smoking. Pimatisiwin.

[32] Kim H., Clark P.I. (2006). Cigarette smoking transition in females of low socioeconomic status: Impact of state, school, and individual factors. J. Epidemiol. Community Health.

[33] Levy D.T., Mumford E.A., Compton C. (2006). Tobacco control policies and smoking in a population of low education women, 1992–2002. J. Epidemiol. Community Health.

[34] McLellan D.L., Kaufman N.J. (2006). Examining the effects of tobacco control policy on low socioeconomic status women and girls: An initiative of the tobacco research network on disparities (trend). J. Epidemiol. Community Health.

[35] Moore R.S., Lee J.P., Antin T.M.J., Martin S.E. (2006). Tobacco free workplace policies and low socioeconomic status female bartenders in San Francisco. J. Epidemiol. Community Health.

[36] Shavers V.L., Fagan P., Jouridine Alexander L.A., Clayton R., Doucet J., Baezconde-Garbanati L. (2006). Workplace and home smoking restrictions and racial/ethnic variation in the prevalence and intensity of current cigarette smoking among women by poverty status, TUS-CPS 1998–1999 and 2001–2002. J. Epidemiol. Community Health.

[37] Moore R.S., Annechino R.M., Lee J.P. (2009). Unintended consequences of smoke-free bar policies for low-ses women in three California Counties. Amer. J. Prev. Med..

[38] Moore R.S., McLellan D.L., Tauras J.A., Fagan P. (2009). Securing the health of disadvantaged women: A critical investigation of tobacco-control policy effects on women worldwide. Amer. J. Prev. Med..

[39] Greaves L., Hemsing N. (2009). Sex, gender, diversity and second-hand smoke policies: Implications for disadvantaged women. Amer. J. Prev. Med..

[40] Greaves L., Johnson J., Qu A., Okoli C.T., Hemsing N., Barney L. (2012). Gender identity, ethnic identity and smoking among first nations adolescents. J. Aboriginal Health.

[41] Torchalla I., Okoli C., Bottorff J.L., Qu A., Poole N., Greaves L. (2012). Smoking cessation programs targeted to women: A systematic review. Women Health.

[42] Action on Smoking and Health (1993). Her Share of Misfortune: Women, Smoking and Low Income.

[43] Simms M., Smith C. (1986). Teenage Mothers and Their Partners: Health and Social Security Research Report 15.

[44] O’Loughlin J., Kishchuk N., DiFranza J., Tremblay M., Paradis G. (2002). The hardest thing is the habit: A qualitative investigation of adolescent smokers’ experience of nicotine dependence. Nicotine Tob. Res..

[45] Amos A., Bostock Y. (2007). Young people, smoking and gender—A qualitative exploration. Health Educ. Res..

[46] Tilleczek K.C., Hine D.W. (2006). The meaning of smoking as health and social risk in adolescence. J. Adolesc..

[47] Jonsdottir R., Jonsdottir H. (2007). The experience of women with advanced chronic obstructive pulmonary disease of repeatedly relapsing to smoking. Scand. J. Caring Sci..

[48] West R., McNeill A., Raw M. (2000). Smoking cessation guidelines for health professionals: An update. Thorax.

[49] Lancaster T., Stead L. (2008). Physician advice for smoking cessation. Cochrane Database Syst. Rev..

[50] Jordan T.R., Dake J.R., Price J.H. (2006). Best practices for smoking cessation in pregnancy: Do obstetrician/gynecologists use them in practice?. J. Womens Health.

[51] Ruger J.P., Lazar C.M. (2012). Economic evaluation of pharmaco-and behavioral therapies for smoking cessation: A critical and systematic review of empirical research. Annu. Rev. Public Health.

[52] Bonevski B., Bryant J., Paul C. (2011). Encouraging smoking cessation among disadvantaged groups: A qualitative study of the financial aspects of cessation. Drug Alcohol Rev..

[53] (2013). Who Report on the Global Tobacco Epidemic,2013: Enforcing Bans on Tobacco Advertising, Promotion and Sponsorship.

[54] Burgess D.J., Fu S.S., van Ryn M. (2009). Potential unintended consequences of tobacco-control policies on mothers who smoke: A review of the literature. Amer. J. Prev. Med..

[55] Healton C.G., Vallone D., Cartwright J. (2009). Unintended consequences of tobacco policies: Implications for public health practice. Amer. J. Prev. Med..

[56] Hemsing N., O’Leary R., Chan K., Okoli C., Greaves L. (2011). Partner support for smoking cessation during pregnancy: A systematic review. Nicotine Tob. Res..

[57] Robinson J., Ritchie D., Amos A., Martin C., Greaves L., Cunningham-Burley S. (2010). “Waiting until they got home”—Gender, smoking and tobacco exposure in households in scotland. Soc. Sci. Med..

[58] Robinson J., Ritchie D., Amos A., Greaves L., Cunningham-Burley S. (2011). Volunteered, negotiated, enforced: Family politics and the regulation of home smoking. Soc. Health Illn..

[59] Phillips R., Amos A., Ritchie D., Cunningham-Burley S., Martin C. (2007). Smoking in the home after the smoke-free legislation in scotland: Qualitative study. BMJ.

[60] Crane C.A., Hawes S.W., Weinberger A.H. (2013). Intimate partner violence victimization and cigarette smoking: A meta-analytic review. Trauma Violence Abus..

[61] Torchalla I., Okoli C.T., Malchy L., Johnson J.L. (2011). Nicotine dependence and gender differences in smokers accessing community mental health services. J. Psychiat. Ment. Health Nurs..

[62] Okoli C.T., Torchalla I., Khara M. (2012). Sex differences in nicotine dependence among addictions clients accessing a smoking cessation programme in Vancouver, British Columbia, Canada. J. Psychiat. Ment. Health Nurs..

[63] Okoli C.T., Khara M., Torchalla I., Ensom M.H., Oliffe J.L., Bottorff J.L., Stanley P.J. (2011). Sex differences in smoking cessation outcomes of a tailored program for individuals with substance use disorders and mental illness. Addict. Behav..

[64] Balbach E.D., Herzberg A., Barbeau E.M. (2006). Political coalitions and working women: How the tobacco industry built a relationship with the coalition of labor union women. J. Epidemiol. Community Health.

[65] Shopland D.R., Anderson C.M., Burns D.M. (2006). Association between home smoking restrictions and changes in smoking behaviour among employed women. J. Epidemiol. Community Health.

[66] Pachankis J.E., Westmaas J.L., Dougherty L.R. (2011). The influence of sexual orientation and masculinity on young men’s tobacco smoking. J. Consult. Clin. Psychol..

[67] Bottorff J.L., Oliffe J.L., Kelly M.T., Greaves L., Johnson J.L., Ponic P., Chan A. (2010). Men’s business, women’s work: Gender influences and fathers’ smoking. Sociol. Health Illn..

[68] Johnson J., Greaves L., Repta R. (2009). Better science with sex and gender: Facilitating the use of a sex and gender-based analysis in health research. Int. J. Equity Health.

[69] Oliffe J.L., Greaves L. (2012). Designing and Conducting Gender, Sex, and Health Research.

[70] Vancouver Coastal Health Women’s Health Committee (2009). Framework for Girls’ and Women Centred Health: An. Implementation Guide for Vancouver Coastal Health.

[71] Johnson J., Ratner P., Malchy L., Okoli C., Procyshyn R., Bottorff J., Groening M., Schultz A., Osborne M. (2010). Gender-specific profiles of tobacco use among non-institutionalized people with serious mental illness. BMC Psychiat..

[72] Abrahams N., Devries K., Watts C., Pallitto C., Petzold M., Shamu S., García-Moreno C. (2014). Worldwide prevalence of non-partner sexual violence: A systematic review. Lancet.

[73] Poole N., Greaves L. (2012). Becoming Trauma Informed.

[74] AWARE Starss (Start Thinking about Reducing Secondhand Smoke). http://www.aware.on.ca/starss.

[75] Stewart M.J., Kushner K.E., Makwarimba E., Spitzer D., Letourneau N.L., Greaves L., Boscoe M. (2010). There’s a way out for me: Insights from support intervention for low-income women who smoke. Womens Health Urban Life.

[76] Stewart M., Greaves L., Kushner K., Letourneau N., Spitzer D., Boscoe M. (2011). Where there is smoke there is stress: Low-income women identify support needs and preferences for smoking reduction. Health Care Women Int..

[77] Horne T., Kirby S., Trudeau J. (1999). Final Evaluation of Catching Our Breath Too.

[78] Barr C.A. (1996). Stopping for Me: Women, Disadvantage and Smoking.

[79] Poole N., Lyon J., Poole N., Greaves L. (2012). Integrating treatment of tobacco with other substances in a trauma-informed way. Becoming Trauma Informed.

[80] Poole N., Greaves L. (2007). Highs and Lows: Canadian Perspectives on Women and Substance Use.

[81] Nathoo T., Poole N., Greaves L. (2013). Women and Tobacco: A Casebook.

[82] Urquhart C., Jasiura F., Poole N., Greaves L. (2012). Collaborative change conversations: Integrating trauma-informed care and motivational interviewing with women. Becoming Trauma Informed.

[83] Lai D., Cahill K., Qin Y., Tang J.L. (2010). Motivational Interviewing for Smoking Cessation; Cochrane Database Syst. Rev..

[84] Covington S.S. (2008). Women and addiction: A trauma-informed approach. J. Psychoactive Drug..

[85] Covington S.S., Burke C., Keaton S., Norcott C. (2008). Evaluation of a trauma-informed and gender-responsive intervention for women in drug treatment. J. Psychoactive Drug..

[86] Gelb K., Pederson A., Greaves L. (2012). How have health promotion frameworks considered gender?. Health Promot. Int..

[87] Pederson A., Ponic P., Greaves L., Mills S., Christilaw J., Frisby W., Humphries K., Poole N., Young L. (2010). Igniting an agenda for health promotion for women: Critical perspectives, evidence-based practice, and innovative knowledge translation. Can. J. Public Health.

[88] Berridge V., Loughlin K. (2005). Smoking and the new health education in Britain. Amer. J. Public Health.

[89] American Cancer Society 1972 Campaign: Smoking is Very Glamorous. http://viralworthy.com/post/396739823/smoking-is-very-glamorous.

[90] Regional Cancer Centre. http://adsoftheworld.com/media/print/regional_cancer_centre_breast?size=original.

[91] Chapman S. (1999). Smoking and women: Beauty before age?. BMJ.

[92] Greaves L. (2014). Can tobacco control be transformative? Reducing gender inequity and tobacco use among vulnerable populations. Int. J. Environ. Res. Public Health.

[93] Greaves L., Tungohan E. (2007). Engendering tobacco control: Using an international public health treaty to reduce smoking and empower women. Tob. Control.

[94] Bosdriesz J., Mehmedovic S., Witvliet M., Kunst A. (2014). Socioeconomic inequalities in smoking in low and mid income countries: Positive gradients among women?. Int. J. Equity Health.

[95] Thun M., Peto R., Boreham J., Lopez A.D. (2012). Stages of the cigarette epidemic on entering its second century. Tob. Control.

[96] Greaves L. (2007). Gender, equity and tobacco control. Health Sociol. Rev..

[97] Poole N., Urquhart C. (2009). Trauma Informed Approaches in Addictions Treatment, Gendering the National Framework Series.

[98] Hemsing N., Greaves L., Greaves L., Pederson A., Poole N. (2014). Igniting global tobacco control. Making It Better: Gender-Transformative Health Promotion.

[99] Tod A.M. (2003). Barriers to smoking cessation in pregnancy: A qualitative study. Brit. J. Community Nurs..

